# Cation-doping strategies for tuning of zirconia acid–base properties

**DOI:** 10.1098/rsos.211423

**Published:** 2022-02-23

**Authors:** Maicon Delarmelina, C. Richard A. Catlow

**Affiliations:** ^1^ School of Chemistry, Cardiff University, Main Building, Park Place, Cardiff CF10 3AT, UK; ^2^ UK Catalysis Hub, Research Complex at Harwell, STFC Rutherford Appleton Laboratory, Didcot, Oxfordshire OX11 0FA, UK; ^3^ Department of Chemistry, University College London, 20 Gordon Street, London WC1 HOAJ, UK

**Keywords:** acid–base properties, biofuels, zirconia, deoxygenation reactions, biofuel upgrading

## Abstract

The role of Y-, Ca- and Ce-doping of cubic zirconia (c-ZrO_2_) (111) surface on its acidity, basicity and the interplay between surface acid–base pairs is investigated by computational methods. The most stable surface structures for this investigation were initially determined based on previous studies of Y-doped c-ZrO_2_ (111) and by a detailed exploration of the most stable configuration for Ca-doped c-ZrO_2_ (111) and Ce-doped c-ZrO_2_ (111). Next, surface mapping by basic probe molecules (NH_3_ and pyridine) revealed a general reduction of the acidity of the surface sites, although a few exceptions were observed for zirconium ions at next nearest neighbour (NNN) positions to the oxygen vacancy and at the nearest neighbour (NN) position to the dopants. Adsorption of CO_2_ over basic sites revealed a cooperative interplay between acid–base groups. In this case, the overall effect observed was the decrease of the calculated adsorption energies when compared with the pristine surface. Moreover, spontaneous formation of *η*^3^-CO_2_ systems from initial *η*^2^-CO_2_ configurations indicates a decrease in the required energy for forming oxygen vacancies in the doped ZrO_2_ systems at NNN positions or further away from the existing vacancy site.

## 1. Introduction

Production of liquid fuels from biomass has gained increasing industrial and academic attention as an alternative energy source to fossil-based fuels [1]. The so-called first-generation biofuels (bioethanol and biodiesel) have been intensively investigated in the last decades; however, their production from food crops (sugar cane, corn, soya beans, etc.) is seen as a drawback to the application of this technology at larger scales. Alternatively, the use of non-edible and underused biomass (waste, wood, algae, etc.), in particular lignocellulosic biomass, has been intensively investigated as an alternative source of biofuels and chemicals [2,3]. Such alternatives represent a promising additional approach to the reduction of greenhouse gas emissions, energy security and a circular economy, without imposing a competition between food and fuel production.

Lignocellulosic biomass is mainly composed of cellulose, hemicellulose and lignin, which after thermochemical treatment can be converted into bio-crude oil, biochar, a gas mixture (CO_2_, CH_4_ and H_2_, depending on the type of biomass and reaction conditions) and an aqueous phase, mainly composed of alcohols, acids and phenols [4,5]. Hydrothermal liquefaction is currently one of the most advantageous method for biomass conversion and both, pre-treated bio-crude oil and the aqueous phase produced via this method, have the potential to be used as sources of transport biofuels and other added-value chemicals. However, prior to their utilization as biofuels, their mixture of organic species requires chemical upgrading to reduce the number of oxygen-containing derivatives responsible for lower energy efficiency, corrosive effects and low chemical stability [3].

Novel heterogeneous catalysts capable of efficiently processing such biomass-derived feedstocks towards the deoxygenation of their components (aldehyde, alcohols, organic acids and esters) have been extensively investigated [6–9]. Acid–base bifunctional catalysts, such as TiO_2_, MnO_2_ and CeO_2_, are particularly desirable in this context, since they are capable of acting as both oxidizing and reducing agents, allowing multiple one-pot transformations [10]. They can also have their acid–base properties tailored by different approaches in order to optimize their activity and selectivity, as was previously observed, for example, in ethanol to n-butanol condensation [11], the production of 5-hydroxymethylfurfural from cellulose [12,13] and the ketonization of organic acids [14–16].

Zirconia (ZrO_2_) is another example of a bifunctional catalyst which has found great interest in the materials science community as a prominent material for wide-ranging industrial applications, including biorefinery processes [8,17–22]. A plethora of modified ZrO_2_ catalysts has been investigated; however, the use of dopants is probably still the most common approach for promoting changes in structure, stability, reactivity and selectivity of these systems. Doping using Ca [23–26], Y [27–31], Ce [32–35] or less widely used cations, such as Fe, Mn, Ti, Sc, Al, Er and alkali metals, among others [36–40], has been largely investigated for different applications. Despite their popularity and the large number of experimental and computational studies previously published, Ca-, Y-, Ce-doped ZrO_2_ systems have not been analysed in detail, particularly concerning the effect of such dopants on the acid–base properties of ZrO_2_.

In this study, we have explored, by Density Functional Theory (DFT) methods, the acid–base properties of pristine and doped cubic zirconia (c-ZrO_2_) (111) surfaces by the adsorption of three probe molecules (CO_2_, NH_3_ and pyridine). This is the first step towards understanding the role of the metal oxide surface itself, before entering the realm of more complicated, but highly relevant, molecular environment under reaction conditions (e.g. hydroxylated surfaces) [41]. Our results not only provide insight into the changes of individual acid and basic surface sites upon doping, but also show how the reactivity of surface acid–base pairs is modified. These findings shed light on the role of dopants on the changes of the amphoteric behaviour of ZrO_2_-based catalysts and its catalytic activity in deoxygenation reactions of bio-oil components.

## 2. Methodology

All calculations were performed using the Vienna *ab initio* simulation package within the framework of periodic Density Funcional Theory (DFT). The electronic structure of all the systems modelled employed the revised Perdew–Burke–Ernzerhof (RPBE) functional combined with Grimme's semiclassical D3 dispersion correction and Coulomb repulsive interaction (*U* = 4 eV) for *d* orbitals of Zr, in accordance with our previous publication [42]. For dopant atoms, the Hubbard correction was only used for *f* orbitals of Ce (*U* = 4.5 eV) [43]; no correction was applied to Ca or Y orbitals. The electron–ionic core interaction was represented by the projector-augmented wave potentials and the cut-off energy was selected after extensive benchmarking and set to 550 eV [42]. The Zr 4s^2^4p^6^4d^2^5s^2^ and O 2s^2^2p^4^ orbitals were explicitly included as valence electrons. Brillouin zone sampling was performed using the Monkhorst–Pack scheme with a k-point grid of 5 × 5 × 1 together with a Gaussian smearing broadening of 0.02 eV. Forces and electronic self-consistent field (SCF) convergence were set at 10^−2^ eV Å^−1^ and 10^−5^ eV, respectively. Dipole corrections were additionally used during all calculations, according to the method by Makov & Payne [44] and Neugebauer & Scheffler [45] The optimized lattice constants obtained at this theory level [42] were used in this work to construct the investigated surface models.

The slab model for the c-ZrO_2_ (111) surface used a 2 × 2 × 3 supercell containing 48 zirconium and 96 oxygen atoms. The resulting structure presented three O-Zr-O trilayers, of which the top two (32 zirconium and 64 oxygen atoms) were allowed to relax in all optimizations. A vacuum box of 15 Å in the *z*-direction was added to the surface in order to avoid undesired interactions between slab images. Further details are given in the electronic supplementary material, figure S1.

All reported adsorption energies (*E*_ads_) were calculated using equation (2.1), where *E*_(Clean Surface)_ is the total energy of the clean surface, *E*_(Adsorbate)_ is the energy of the adsorbate in the 15 Å × 15 Å × 15 Å vacuum box and *E*_(Surface + Adsorbate)_ is the energy of the surface interacting with the adsorbate.

2.1 $$E_{\mathrm{ads}}\;=\;E_{\left(\mathrm{Surface}+\mathrm{Adsorbate}\right)}\text{–}(E_{\left(\mathrm{Clean}\;\mathrm{Surface}\right)}\;+\;E_{(\mathrm{Adsorbate})}).$$

## 3. Results

### 3.1. Pristine c-ZrO_2_ (111) surface

**Figure 1.**
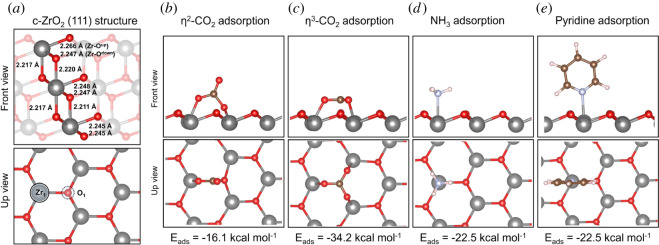
Structure of c-ZrO_2_ (111) system (*a*) and calculated adsorption energies (*E*_ads_) for ((*b*) and (*c*)) CO_2_, (*d*) NH_3_ and (*e*) pyridine.

The pristine c-ZrO_2_ (111) surface is composed of distinct Zr-O^up^ and Zr-O^down^ ion pairs, in which only the former can interact with any adsorbed substrate; the latter is localized in the subsurface. For Zr-O^up^ an optimized bond length of 2.266 Å was computed, whereas for Zr-O^down^ the obtained bond length was 2.247 Å. Other relevant bond lengths are shown in figure 1*a*. Overall, the evaluation of acid and basic properties of c-ZrO_2_ (111) surface was the simplest case investigated in this work, having only one type of acid site and one type of basic site (Zr_1_ and O_1_, figure 1*a*). For these cases, the adsorption of the set of probing molecules (CO_2_, NH_3_ and pyridine) was considered to estimate the acidity and basicity of these surface sites (figure 1*b–e*).

The adsorption of CO_2_ was calculated to occur via interaction of the carbon atom with a surface oxygen atom (O_1_) and formation of a new C-O bond with length of 1.420 Å. The resulting carbonate species can either remain perpendicular to the surface, formally interacting in a bidentate fashion (*η*^2^-CO_2_, figure 1*b*), or rearrange to a parallel orientation to the surface, resulting in the formation of a carbonate anion adsorbed in a threefold type of interaction (*η*^3^-CO_2_, figure 1*c*). The computed adsorption energies for these cases were, respectively, −16.1 and −34.2 kcal mol^−1^ for *η*^2^-CO_2_ and *η*^3^-CO_2_. The latter configuration is significantly more stable than the former, and it has been previously used to assign experimentally observed IR bands around 1400 cm^−1^ as C-O stretching modes, detected after pre-treatment of ZrO_2_ samples at high temperatures [46].

The interaction of NH_3_ and pyridine to the acid site Zr_1_ led to very similar results, with calculated adsorption energies of −22.5 kcal mol^−1^ for both probes.

### 3.2. Doped c-ZrO_2_ (111) surface

Initially, the substitution of Zr ions by the selected dopant was investigated to determine the most stable configuration for these systems, considering the possibility of the dopant being located at surface or subsurface sites, as well as carefully evaluating the most appropriate location of compensating oxygen vacancies. The surface structure of Y-doped c-ZrO_2_ (111) has been thoroughly investigated in previous works. Xia *et al.* [29,31] used interatomic potentials to investigate Y-stabilized c-ZrO_2_. The authors reported that in these systems, two Y^3+^ cations will preferably be located close to each other, whereas the created oxygen vacancies will occupy the next nearest neighbour (NNN) sites. Moreover, it was observed that one of these cations will be located at the (111) surface and the oxygen vacancy at the subsurface. Similar results were later obtained by Cadi-Essadek *et al.* [28,30,47] and Ricca *et al.* [48] using DFT approaches (generalized gradient approximation (GGA) and hybrid functionals). It is worth noticing that alternative configurations have also been identified by Chaopradith *et al.* [49], which may be appropriate for higher dopant concentrations. The effect of dopant and vacancy distribution on sorption energies will be a topic for future study.

**Figure 2.**
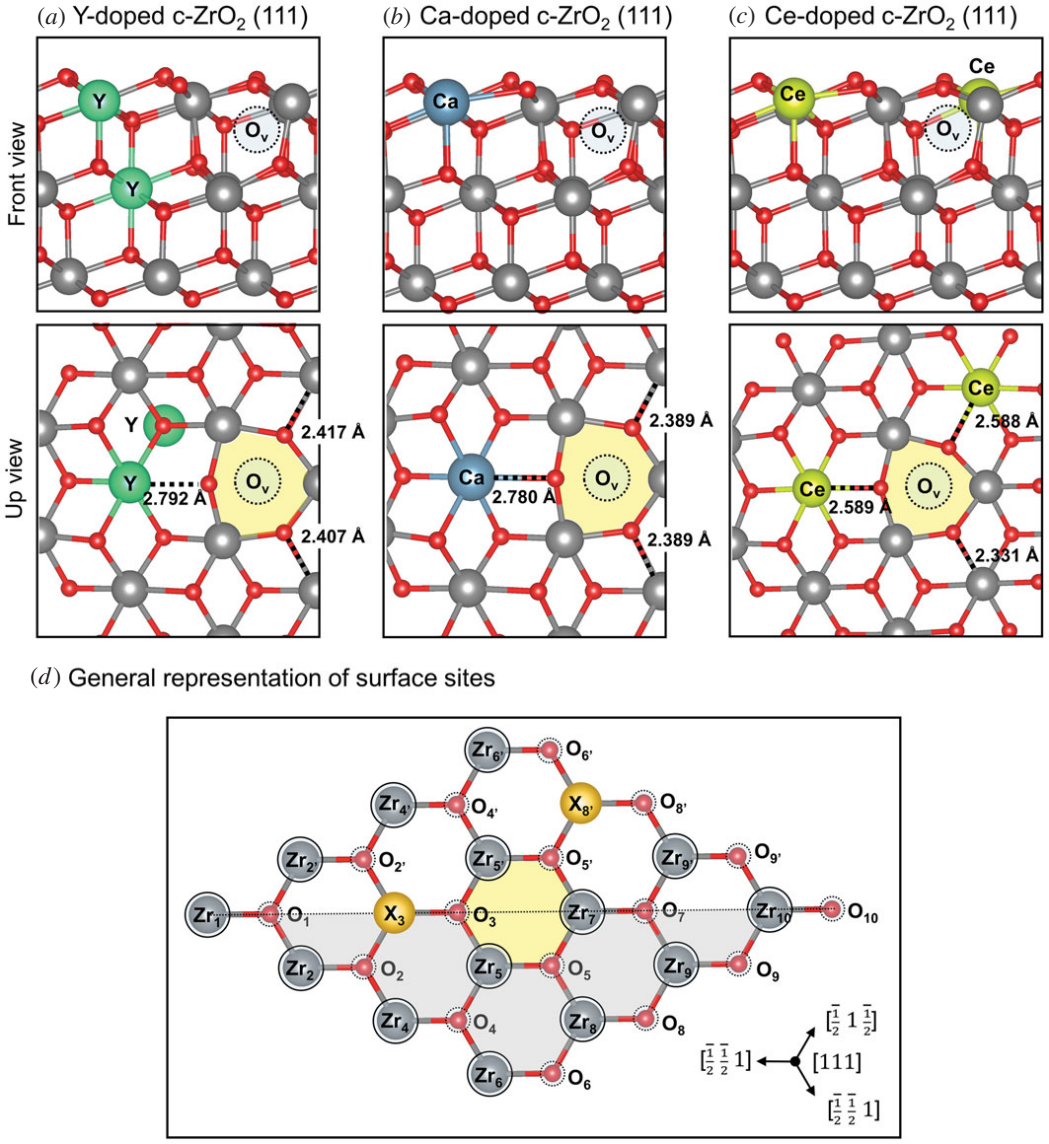
Selected configuration for (*a*) Y-, (*b*) Ca-, (*c*) Ce-doped c-ZrO_2_ (111) and general representation of surface sites of the doped systems (*d*). Yellow hexagon indicates the position of subsurface oxygen vacancy.

In the present study, the Y-doped c-ZrO_2_ (111) model (figure 2*a*) has two Y ions at the nearest neighbour (NN) position and one oxygen vacancy in the subsurface and at the NNN position to both Y ions, as reported by Ricca *et al*. [48]; also here, we observed significant cation-oxygen bond elongation around the vacancy site. For these cases, Y-O bond lengths varied roughly from 2.4 to 2.8 Å, whereas Zr-O bond lengths varied from 2.2 to 2.4 Å (figure 2*a*).

The structure of Ca-doped c-ZrO_2_ systems has been extensively debated using various experimental characterization techniques [50]. While X-ray diffraction studies of Morinaga *et al.* [51] suggested the oxygen vacancies in such systems would be preferably located at the NN position to the calcium ion, later investigations by extended X-ray absorption fine structure (EXAFS) spectroscopy strongly support models in which the vacancy is located at the NNN position [52,53]. Here, we have determined the most stable structure for Ca-doped c-ZrO_2_ (111) systems by evaluating alternative positions for the replacement of one Zr ion by one Ca ion with the creation of a compensating oxygen vacancy. The details for this preliminary investigation are described in the electronic supplementary material, figure S2. Overall, our findings show that the most stable system is that in which Ca and O vacancy are located at the NNN position and segregated to the (111) surface (figure 2*b*), in agreement with the results from EXAFS spectroscopy. As was calculated for the Y-doped c-ZrO_2_ (111) system, also here the cation-oxygen bonds around the vacancy site were observed to elongate significantly, with Ca-O bond lengths varying roughly from 2.5 to 2.8 Å and Zr-O bond lengths varying from 2.2 to 2.4 Å. Recently, de Souza & Appel [24] investigated oxygen vacancy formation in the Ca-doped m-ZrO_2_ (-111) surface, suggesting that the preferential localization of the formed O vacancy is neighbouring the dopant [24]. In this work on c-ZrO_2_, when such a possibility was considered, one of the surrounding oxygens always moved towards the dopant during optimization, in order to fill its coordination sphere (see electronic supplementary material, figure S2).

Despite the large number of publications regarding ZrO_2_/CeO_2_, the most commonly investigated systems are bulk structures [33,54–56], surface models with large cerium contents [43,57–62] or stoichiometric structures only [63,64]. To the best of our knowledge, detailed investigations of Ce-doped c-ZrO_2_ (111) structure at low dopant concentrations have not been reported. In this investigation, the most stable structure for this system was determined by replacing two Zr ions by two Ce ions, which we assume are reduced to Ce^3+^ with the creation of an oxygen vacancy, and by considering these species at surface and subsurface sites. Details for this preliminary investigation are described in the electronic supplementary material, table S1. To ensure the formation of reduced Ce^3+^ species in the system, all structures had Ce atoms temporarily replaced by La atoms for pre-optimization of the ionic structure of the system [65,66]. The larger radii of La led to an elongation of the dopant-oxygen bonds, biasing that dopant site to form a Ce^3+^ species in the next step of our methodology. After the pre-optimizations, the Ce atoms were returned to the structure and were reoptimized, while a triplet-state electronic structure was imposed to the system. The most stable system identified here was that in which both Ce ions and O vacancy are segregated to the surface, with these species at the NNN position from each other (figure 2*c*). Maleki & Pacchioni [38] have recently explored isovalent dopants on t-ZrO_2_ (101) surface, and their results when replacing one Zr by one Ce atom have also shown preferential segregation of this dopant to the surface [38].

After determining the most stable configuration for the Y-, Ca- and Ce-doped systems constructed here, the changes in the acid–base properties of the surface sites were mapped by probe molecules. It is worth mentioning that not only the neighbouring sites to the dopant were considered but all surface sites present in our model (figure 2*d*). In this way, we hope to achieve a better understanding of how such surfaces may have their reactivity affected by these dopants, especially at low-dopant concentrations.

### 3.3. Probing acid sites on doped c-ZrO_2_ (111) surface: NH_3_ and pyridine adsorption

**Figure 3.**
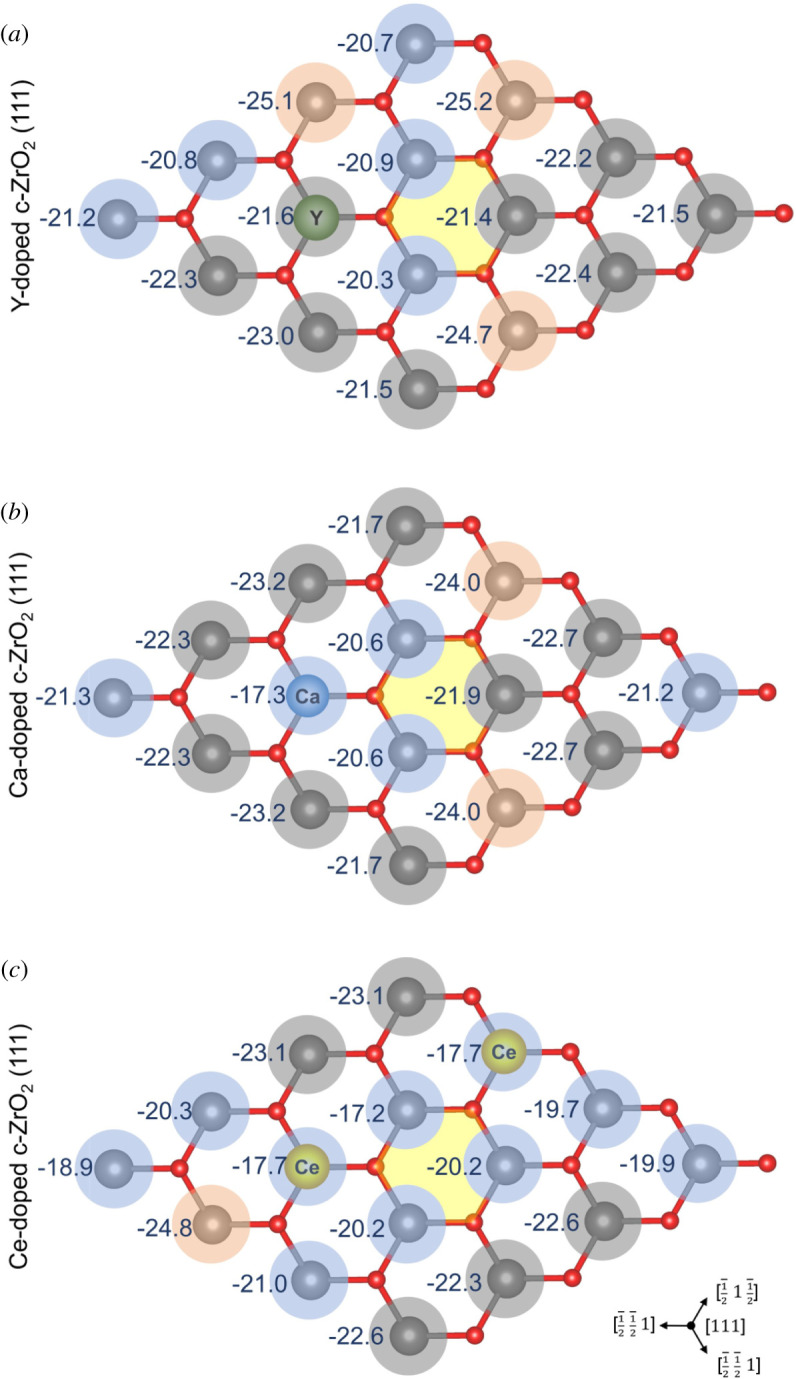
Calculated NH_3_ adsorption energies over distinct acid sites of (*a*) Y-, (*b*) Ca- and (*c*) Ce-doped ZrO_2_ (111) systems. Values are given in kcal mol^−1^. Yellow hexagon indicates the position of subsurface oxygen vacancy. Coloured circles illustrate the comparison of calculated adsorption energies to that of the pristine surface (reference value for NH_3_ adsorption: 22.5 kcal mol^−1^). *Red:* increase of calculated Lewis acidity; *grey:* energy variation smaller than ±1 kcal mol^−1^; *blue:* decrease of calculated Lewis acidity.

The Lewis acidity of the surface sites was initially probed by the adsorption of NH_3_ molecules at the on-top position of each zirconium or dopant site. Calculated adsorption energy values are shown in figure 3 and the coloured circles over each site illustrate the variation observed when these values are compared with the pristine c-ZrO_2_ (111) surface (figure 1*d*, *E*_ads_ (NH_3_) = 22.5 kcal mol^−1^): *red circles* indicate an increase of calculated Lewis acidity; *grey circles* are used when the calculated energy variation is smaller than ±1 kcal mol^−1^; *blue circles* indicate a decrease of calculated Lewis acidity. Calculated values are also available in the electronic supplementary material, tables S2 and S4.

The calculated adsorption energies ranged between −20.3 and −25.2 kcal mol^−1^ for Y-doped system, −17.3 and −24.0 kcal mol^−1^ for Ca-doped system, and −17.2 and −24.8 kcal mol^−1^ for Ce-doped ZrO_2_ (111) (figure 3*a*–*c*). Overall, we observe that very few sites had their Lewis acidity increased by doping. In fact, most of the surface sites considered here showed a decrease of their Lewis acidity or remained roughly unchanged when compared with the pristine system. Such results are in agreement with the previously described reduction of ZrO_2_ acidity upon doping by the same ions [11,14–16,24,67,68].

Interestingly, the dopant sites Ca and Ce (as illustrated in figure 3*b,c*) showed a significant reduction of their adsorption energies compared with that of the pristine system (−17.3 and −17.7 kcal mol^−1^, respectively) and they are the acid site with the smallest Lewis acidities within such systems, although the Zr sites localized between the two Ce atoms in figure 3*c* have a similar computed adsorption energy of −17.2 kcal mol^−1^. On the other hand, the dopant site Y (figure 3*a*) showed a variation of only +0.9 kcal mol^−1^ when compared with the pristine system. For this case, the acid sites with the smallest Lewis acidity were those neighbouring the vacancy site, with computed adsorption energies ranging between −20.3 and −20.9 kcal mol^−1^.

Finally, the higher Lewis acidities were observed for Zr sites at the NNN position to the oxygen vacancy sites in the Ca-doped system (figure 3*b*, *E*_ads_ (NH_3_): −24.0 kcal mol^−1^), Zr sites at the NN position to the dopant in Ce-doped system (figure 3*c*, *E*_ads_ (NH_3_): −24.8 kcal mol^−1^) and at both the NNN position to the oxygen vacancy and the NN position to the dopant in Y-doped system (figure 3*a*, *E*_ads_ (NH_3_): −24.7, −25.2 and −25.1 kcal mol^−1^).

**Figure 4.**
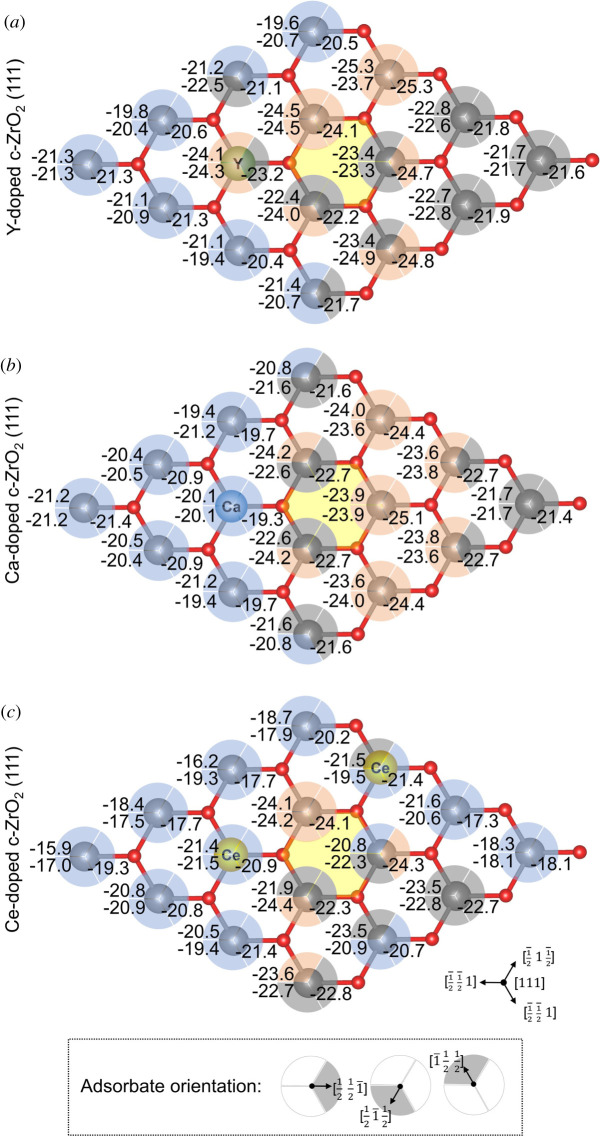
Calculated pyridine adsorption energies over distinct acid sites of (*a*) Y-, (*b*) Ca- and (*c*) Ce-doped ZrO_2_ (111) systems. Values are given in kcal mol^−1^. Yellow hexagon indicates the position of subsurface oxygen vacancy. Coloured partial circles represent the pyridine adsorption site (pyridine-metal and distinct ortho-CH • • • O interactions; see insert) and illustrate the comparison of calculated adsorption energies to that of the pristine surface (reference value for pyridine adsorption: 22.5 kcal mol^−1^). *Red:* increase of calculated Lewis acidity; *grey:* energy variation smaller than ±1 kcal mol^−1^; *blue:* decrease of calculated Lewis acidity.

Next, the doped surfaces were additionally characterized by the adsorption of pyridine molecules. This probe molecule adsorbs to the surface via two distinct interactions: N-metal and ancillary ortho-CH • • • O interactions. Since each acid site is surrounded by three oxygen atoms, three distinct configurations were considered for the adsorption of pyridine, as represented by the partial circles in figure 4. Similar to NH_3_, each partial circles illustrate the variation observed when these values are compared with the pristine c-ZrO_2_ (111) surface (figure 1*e*, *E*_ads_ (pyridine) = 22.5 kcal mol^−1^): *red partial circles* indicate an increase of calculated Lewis acidity; *grey partial circles* are used when the calculated energy variation is smaller than ±1 kcal mol^−1^; *blue partial circles* indicate decrease of calculated Lewis acidity. Calculated values are also available in the electronic supplementary material, tables S5 and S7.

In this case, the calculated adsorption energies ranged between −19.4 and −25.3 kcal mol^−1^ for Y-doped system, −19.3 and −25.1 kcal mol^−1^ for Ca-doped system and −15.9 and −24.4 kcal mol^−1^ for Ce-doped ZrO_2_ (111) (figure 4*a*–*c*). Interestingly, the patterns of increase and decrease of adsorption energies are significantly different from those observed for NH_3_ adsorption, which highlights the importance of not only probing individual acid surface sites, but also the surface metal-oxygen acid–base pairs of amphoteric materials. Furthermore, it shows that the influence of the basic sites, even if by simple forming CH • • • O dipole–dipole interactions, can significantly affect the adsorption energies of this probe. It is likely that the basicity of the surface oxygens involved in such pyridine adsorption is increased by the presence of the dopant and vacancy, contributing to a more exothermic adsorption energy of pyridine—a highly relevant observation since these sites will act synergistically during deoxygenation reactions, which will be further evaluated in the later subsection focusing on the probing of the basic sites by CO_2_ molecules.

Despite the differences computed between NH_3_ and pyridine adsorption energies, the overall role of the dopants remains the same, since most of the acid surface sites showed a decrease or very similar adsorption energies to that of the pristine surface (figure 1*e*). Nevertheless, in a few cases, an increase of acidity was also observed, mainly at the NN and NNN acid sites to the vacancy.

Once again, adsorption at the dopant sites Ca and Ce resulted in a reduction of the calculated adsorption energies for pyridine, when compared with the pristine surface (figure 4*b,c*), whereas Y gave adsorption energies *ca* 2–4 kcal mol^−1^ more exothermic than those computed for Ca and Ce (figure 4*a*).

Interestingly, for both NH_3_ and pyridine adsorption, Ce-doped c-ZrO_2_ (111) was the surface with the largest number of weaker acid sites (*blue circles*, figures 3*c* and 4*c*), followed by Ca-doped (figures 3*b* and 4*b*) and Y-doped c-ZrO_2_ (111) (figures 3*a* and 4*a*). When examining the number of stronger acid sites (*red circles*, figures 3 and 4), Ce-doped c-ZrO_2_ (111) had the smallest number (figures 3*c* and 4*c*). For Ca- and Y-doped c-ZrO_2_ (111), the number of stronger acid sites was roughly the same, although the latter gave computed adsorption energies slightly more exothermic than the former.

### 3.4. Probing basic sites on doped c-ZrO_2_ (111) surface: CO_2_ adsorption

**Figure 5.**
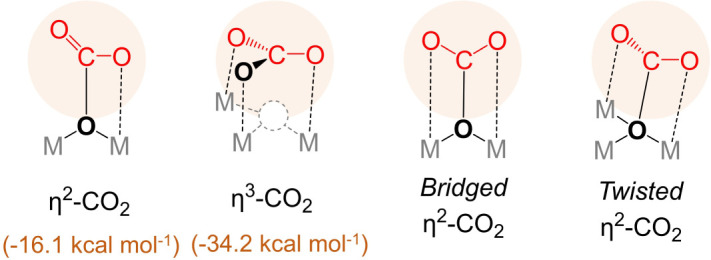
Distinct configurations obtained for CO_2_ adsorption. Calculated adsorption energies over pristine surface are given in parentheses.

**Figure 6.**
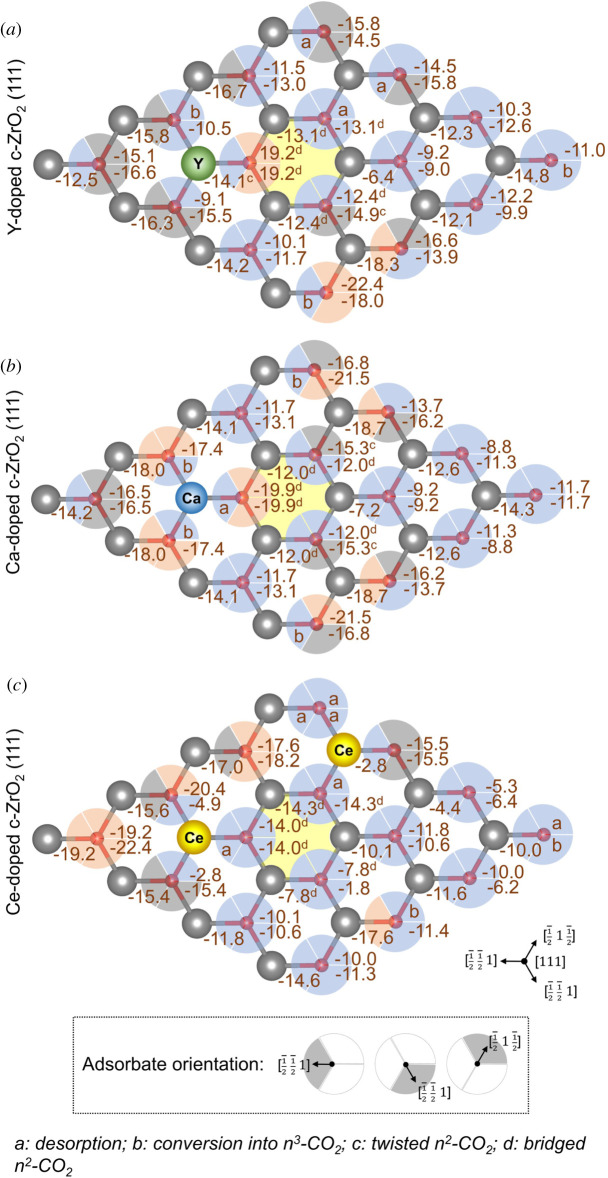
Calculated *η*^2^-CO_2_ adsorption energies over distinct basic sites of (*a*) Y-, (*b*) Ca- and (*c*) Ce-doped ZrO_2_ (111) systems. Values are given in kcal mol^−1^. Yellow hexagon indicates the position of subsurface oxygen vacancy. Coloured partial circles represent the CO_2_ adsorption sites (surface oxygen-C(O_2_) and distinct metal-O(CO) interactions; see insert) and illustrate the comparison of calculated adsorption energies to that of the pristine surface (reference value for *η*^2^-CO_2_ adsorption: 16.1 kcal mol^−1^). *Red:* increase of calculated Lewis basicity; *grey:* energy variation smaller than ±1 kcal mol^−1^; *blue:* decrease of calculated Lewis basicity.

**Figure 7.**
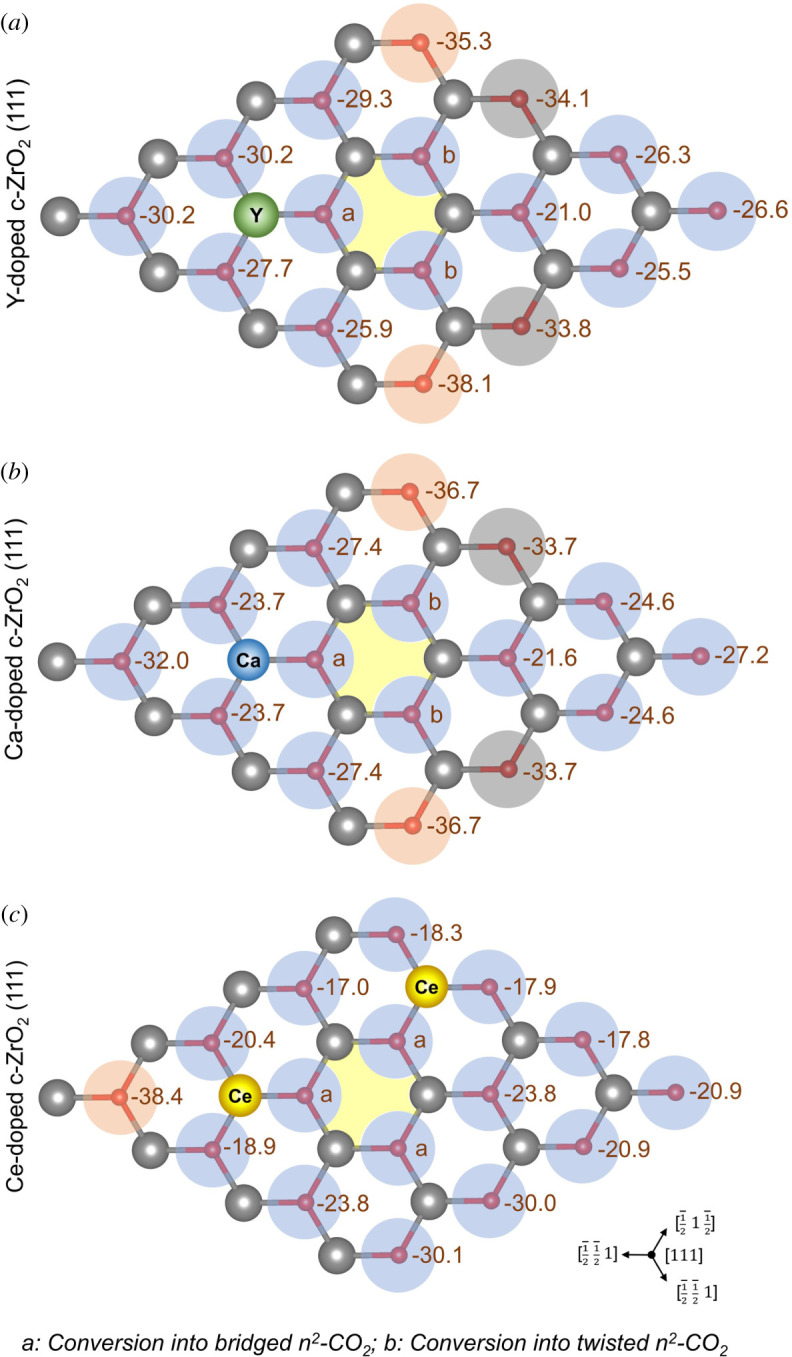
Calculated *η*^3^-CO_2_ adsorption energies over distinct basic sites of (*a*) Y-, (*b*) Ca- and (*c*) Ce-doped ZrO_2_ (111) systems. Values are given in kcal mol^−1^. Yellow hexagon indicates the position of subsurface oxygen vacancy. Coloured circles represent the CO_2_ adsorption sites and illustrate the comparison of calculated adsorption energies to that of the pristine surface (reference value for *η*^3^-CO_2_ adsorption: 34.2 kcal mol^−1^). *Red:* increase of calculated Lewis basicity; *grey:* energy variation smaller than ±1 kcal mol^−1^; *blue:* decrease of calculated Lewis basicity.

Two distinct adsorption modes for CO_2_ were initially considered, according to the results obtained for the pristine surface, *η*^2^- and *η*^3^-CO_2_ modes (figure 1*b,c*), in which the latter corresponds to a threefold type of interaction between the formed carbonate species and the surface. However, two additional configurations were observed for surface sites localized around the vacancy site—bridged *η*^2^-CO_2_ and twisted *η*^2^-CO_2_, as illustrated in figure 5 and indicated in figures 6 and 7. Calculated values are also available in the electronic supplementary material, tables S8 and S13. Considering all *η*^2^-CO_2_ modes, the calculated adsorption energy values varied significantly, ranging between −6.4 and −22.4 kcal mol^−1^ for Y-doped, −7.2 and −21.5 kcal mol^−1^ for Ca-doped, and −1.8 and −22.4 kcal mol^−1^ for Ce-doped c-ZrO_2_ (111) system. Although an increase in the basicity of such systems is expected after doping [11,14–16,24,67,68], the interplay between acid–base strengths involved in the CO_2_ adsorption reveals a more complex picture, similar to that described for the pyridine adsorption. Most of the surface sites considered here for forming *η*^2^-CO_2_ either showed a decrease of the calculated adsorption energies when compared with the pristine system (*blue partial circles*, figure 6) or they were only slightly affected (*grey partial circles*, figure 6). The only cases in which an increase of the calculated *η*^2^-CO_2_ adsorption energies was observed are surface O atoms at NN and NNN positions to both vacancy and/or dopant sites in Y-doped and Ce-doped systems (*red partial circles*, figure 6*a,c*), and surface O atoms coordinated to the dopant and at the NNN position to the vacancy in the Ca-doped c-ZrO_2_ (111) system (*red partial circles*, figure 6*b*).

All doped systems had at least two sites in which O surface sites spontaneously formed *η*^3^-CO_2_ configurations during optimization, instead of the initial *η*^2^-CO_2_ configuration (figure 6*a*–*c*). This behaviour seems to indicate a decrease in the vacancy formation energy of such systems upon doping. The same effect has been previously reported for Ca-doped ZrO_2_ systems during CO_2_ activation [24]. Finally, the formation of *η*^3^-CO_2_ was systematically explored for all basic sites in the doped surfaces considered here (figure 7). Most surface sites showed a reduction of the computed adsorption energies when compared with the pristine system. Interestingly, this same *η*^3^-CO_2_ configuration could not be obtained for those sites neighbouring the O vacancy. For these cases, all attempts to remove these surface O atoms from the lattice led to the restoration of its position and retention of either a bridged or twisted *η*^2^-CO_2_ configuration. Nevertheless, sites at the NNN position or further away from the vacancy site were observed to form *η*^3^-CO_2_ systems spontaneously.

The increasing trends of spontaneous *η*^3^-CO_2_ formation observed for the doped systems indicate that basic surface sites can play a role in the reaction of organic molecules, not only as adsorption sites, but also by being incorporated into the adsorbed intermediates. Such behaviour can be highly relevant, for instance, during transformations involving carbonyl-containing molecules, which could go through a nucleophilic attack by surface oxygens, producing unconventional geminal diol intermediates. Moreover, according to these findings, the adsorption of oxygenated molecules such as alcohols and organic acids over doped ZrO_2_ systems will probably occur at those sites in which an increase in *η*^2^-CO_2_ adsorption energies were computed, since they contain acid–base pairs with the largest strength for interacting with the oxygenated groups and concertedly abstracting the available H^+^ species.

## 4. Summary and conclusion

We have mapped the changes in the strength of acid and basic surface sites of pristine and doped c-ZrO_2_ (111) surface by CO_2_, NH_3_ and pyridine adsorption. Initially, we explored the most stable configurations for the doped surfaces. Although Y-doped c-ZrO_2_ (111) have been previously described, further investigation of Ca- and Ce-doped c-ZrO_2_ (111) systems was required. The most stable structures identified for Y-, Ca- and Ce-doped c-ZrO_2_ (111) surfaces were composed of (i) two Y ions at the NN position, in which only one is located at the surface, and one oxygen vacancy in the subsurface at the NNN position to both Y ions, (ii) one Ca ion and an O vacancy located at the NNN position and segregated to the (111) surface, and (iii) two Ce ions and O vacancy segregated to the surface and at the NNN position from each other, although as noted, alternative configurations are possible.

Calculated adsorption energies of NH_3_ and pyridine showed that most of the acid sites in the doped surfaces had their acidity reduced or only slightly altered when compared with the pristine system. Exceptions were observed during NH_3_ adsorption, in which the zirconium site at the NNN position to the oxygen vacancy and at NN sites to the dopant showed an increase of the calculated adsorption energies. For pyridine adsorption, however, such a comparison was complicated by the influence of the ancillary ortho-CH • • • O interaction and the changing basicity of the surface oxygens, resulting in an unexpected increase of the computed adsorption energies for this molecule. Interestingly, the mapping of such acid–base pairs shows how cooperative interplay between these groups might affect the adsorption of oxygenated bio-oil components under reaction conditions. The preferential adsorption of oxygenated molecules is more probable at an acid–base pair with stronger cooperative interplay between these groups, rather than at stronger basic sites surrounded by weak acid sites.

Probing of basic sites by adsorption of CO_2_ molecules also revealed a cooperative interplay of acid–base pairs in which the expected increase of basicity in doped ZrO_2_ systems was not generally found. Instead, a reduction of CO_2_ adsorption energies was observed for the doped systems. Additionally, an increase in the spontaneous formation of *η*^3^-CO_2_ systems from initial *η*^2^-CO_2_ configurations was calculated for all doped systems, indicating a decrease in the required energy for forming oxygen vacancies. On the other hand, for O sites surrounding the vacancy site, the formation of *η*^3^-CO_2_ systems was not observed. Once again, such insights are highly relevant to the rationalization of the reactivity and selectivity of these catalysts in deoxygenation reactions of biofuel components. First, the detailed screening of the strength of surface acid–base pairs for CO_2_ adsorption can be extended to the identification of the most probable adsorption site, for instance, of protic molecules, such as acetic acid and alcohol, since their adsorption at acid sites can be significantly affected by the neighbouring basic site capable of promoting their deprotonation. Second, the proposed spontaneous formation of *η*^3^-CO_2_ suggests that these O sites may also act as nucleophiles under reaction conditions, leading to the formation of unexpected intermediate species.

Overall, these findings provide a clear picture of the effect of Y, Ca and Ce dopants over the acidity, basicity and the changes in the strength of acid–base pairs of c-ZrO_2_ (111) surface. Moreover, this investigation provides comprehensive insights into the amphoteric behaviour of ZrO_2_-based catalysts in deoxygenation reactions of biofuel components and the expected changes caused by doping and O vacancy formation.

## Data accessibility

The data are provided in the electronic supplementary material [69].
